# Cooking and Season as Risk Factors for Acute Lower Respiratory Infections in African Children: A Cross-Sectional Multi-Country Analysis

**DOI:** 10.1371/journal.pone.0128933

**Published:** 2015-06-04

**Authors:** Hannes Buchner, Eva A. Rehfuess

**Affiliations:** Institute for Medical Informatics, Biometry and Epidemiology, Ludwig-Maximilians-University, Munich, Germany; University Children's Hospital Basel, SWITZERLAND

## Abstract

**Background:**

Acute lower respiratory infections (ALRI) are a leading cause of death among African children under five. A significant proportion of these are attributable to household air pollution from solid fuel use.

**Methods:**

We assessed the relationship between cooking practices and ALRI in pooled datasets of Demographic and Health Surveys conducted between 2000 and 2011 in countries of sub-Saharan Africa. The impacts of main cooking fuel, cooking location and stove ventilation were examined in 18 (n = 56,437), 9 (n = 23,139) and 6 countries (n = 14,561) respectively. We used a causal diagram and multivariable logistic mixed models to assess the influence of covariates at individual, regional and national levels.

**Results:**

Main cooking fuel had a statistically significant impact on ALRI risk (p<0.0001), with season acting as an effect modifier (p = 0.034). During the rainy season, relative to clean fuels, the odds of suffering from ALRI were raised for kerosene (OR 1.64; CI: 0.99, 2.71), coal and charcoal (OR 1.54; CI: 1.21, 1.97), wood (OR 1.20; CI: 0.95, 1.51) and lower-grade biomass fuels (OR 1.49; CI: 0.93, 2.35). In contrast, during the dry season the corresponding odds were reduced for kerosene (OR 1.23; CI: 0.77, 1.95), coal and charcoal (OR 1.35; CI: 1.06, 1.72) and lower-grade biomass fuels (OR 1.07; CI: 0.69, 1.66) but increased for wood (OR 1.32; CI: 1.04, 1.66). Cooking location also emerged as a season-dependent statistically significant (p = 0.0070) determinant of ALRI, in particular cooking indoors without a separate kitchen during the rainy season (OR 1.80; CI: 1.30, 2.50). Due to infrequent use in Africa we could, however, not demonstrate an effect of stove ventilation.

**Conclusions:**

We found differential and season-dependent risks for different types of solid fuels and kerosene as well as cooking location on child ALRI. Future household air pollution studies should consider potential effect modification of cooking fuel by season.

## Introduction

Acute lower respiratory infections (ALRI), responsible for around 845 000 deaths among children under five years of age in the year 2010 [1], are one of the most important causes of death during childhood. The situation in sub-Saharan Africa is especially grave with around 378 000 ALRI deaths occurring in this region alone [2]. Among the reasons for the continued high burden attributable to child ALRI are exposures to a range of risk factors, in particular malnutrition and household air pollution from solid fuel use (HAP), and poor healthcare systems that limit demand for and access to appropriate treatment.

Indeed, children’s exposure to HAP has been associated with a nearly two-fold increase in the odds of suffering from ALRI [3]. In addition, HAP is a known risk factor for chronic obstructive pulmonary disease, cataract, cardiovascular disease and cancer among adults, and was responsible for 3.5 million global deaths in the year 2010 [4]. In Sub-Saharan Africa, it was ranked the second most important risk factor behind malnutrition [4]. HAP exerts a particularly heavy toll among children, with 466 000 HAP-attributable ALRI deaths and more than 40 million HAP-attributable ALRI DALYs (disability-adjusted life years) [5].

In Sub-Saharan Africa, 77% (95% confidence interval: 74%, 81%) of households relied on solid fuels for cooking in the year 2010 [6], and this figure has remained largely unchanged since the 1980s [6,7]. Burning solid fuels on open fires or simple stoves produces high concentrations of hundreds of pollutants, including particulate matter (PM), carbon monoxide (CO), nitrogen oxides and various known carcinogens, such as polyaromatic hydrocarbons and benzene [8]. In these settings, concentrations of health-relevant PM_2.5_ (particulate matter of less than 2.5 micrometres in diameter) can exceed the annual WHO guideline limits of 10 μg/m^3^ by up to two orders of magnitude [9].

Obtaining accurate measurements of PM_2.5_ or PM_10_ in households in developing countries is logistically and methodologically challenging, and many epidemiological studies have therefore relied on household solid fuel use as a proxy measure, including in cross-sectional studies based on routine survey data [10–12]. A problem with survey-based studies that attempt to estimate the impact of HAP exposure on health outcomes is insufficient adjustment for confounding and complex interactions between multiple risk factors [13,3]. Furthermore, the reliance on maternal recall of child symptoms rather than physician- or field worker-based diagnosis makes a distinction between less frequent but severe ALRI and much more frequent but mostly harmless acute upper respiratory infections (AURI) difficult; consequently, ALRI identified through maternal recall is likely to introduce over-reporting and an important bias towards the null.

Reacting to these challenges, the primary objective of this study was to assess solid fuel use for cooking as a risk factor for child ALRI in sub-Saharan Africa, pooling multiple datasets to overcome sample size limitations and taking into account confounders and competing risk factors in a systematic way. The secondary objective was to investigate the specific role of cooking location and stove ventilation on child ALRI.

## Materials and Methods

### Data and study population

**Fig 1:**
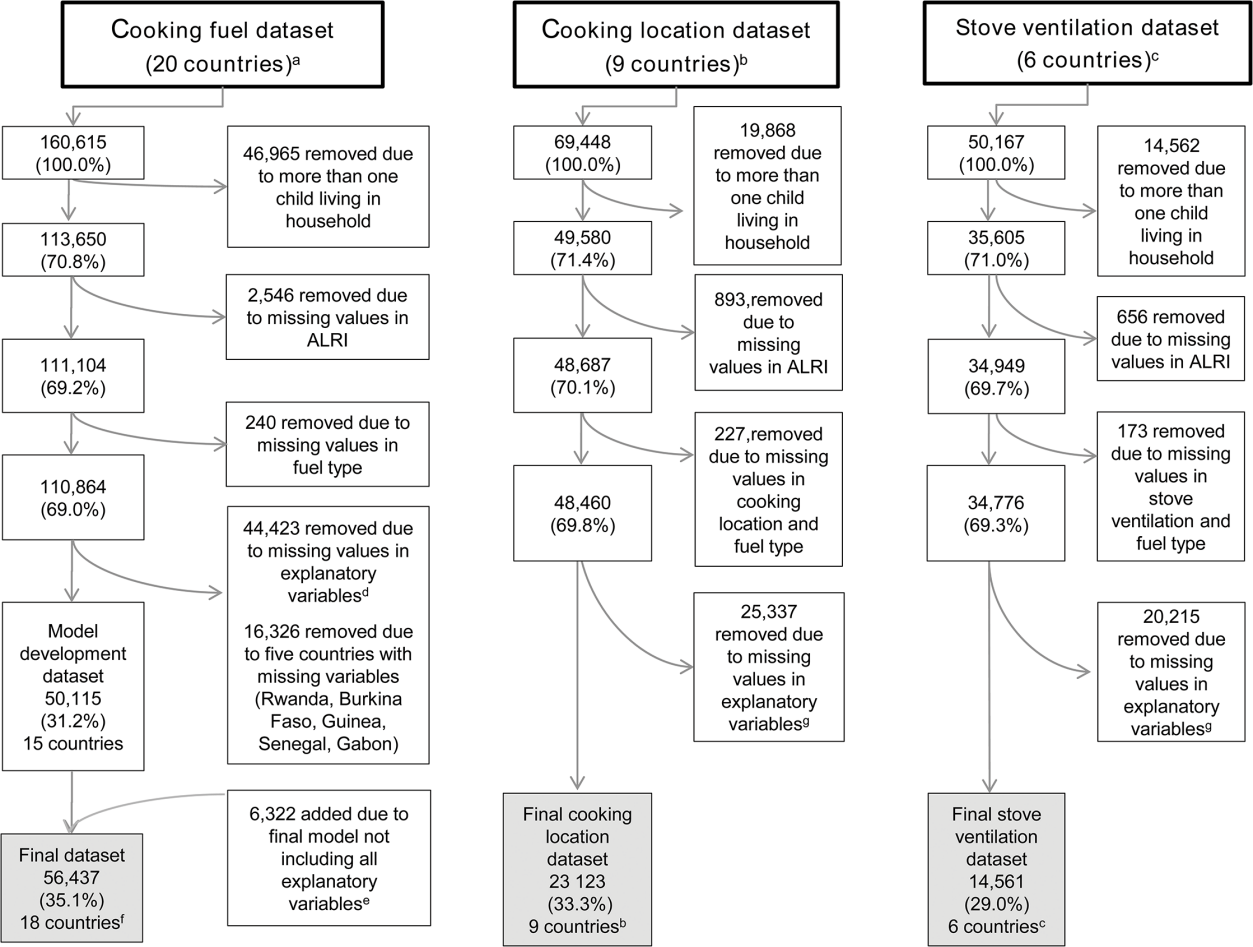
Flow diagram of pooled DHS dataset illustrating where countries and observations are lost due to missing variables or values in analysis of (a) main cooking fuel, (b) cooking location and (c) stove ventilation. ^a^ Benin, Burkina Faso, Cameroon, Ethiopia, Gabon, Ghana, Guinea, Kenya, Madagascar, Mali, Malawi, Mozambique, Namibia, Niger, Rwanda, Senegal, Tanzania, Uganda, Zambia, and Zimbabwe. ^b^ Ethiopia, Ghana, Kenya, Madagascar, Malawi, Namibia, Uganda, Zambia, and Zimbabwe. ^c^ Ghana, Madagascar, Malawi, Namibia, Uganda, and Zambia. ^d^ Missing values in explanatory variables: Maternal smoking, crowding, time to nearest water source, shelter index, wealth index, vaccination index, paternal education, maternal education, possession of health card, child sex, child age, birth order, breastfeeding duration, stunting, geographic location, sex of household head, age of household head. ^e^ Variables not selected in final model: Crowding, wealth index, possession of health card, breastfeeding duration, sex of household head, age of household head. ^f^ Three countries could be added because crowding was not selected in the final model and it was the only missing variable in Burkina Faso, Guinea, and Senegal. ^g^ Missing values in explanatory variables: Maternal smoking, time to nearest water source, shelter index, paternal education, maternal education, child sex, child age, birth order, stunting, vaccination index, geographic location.

Demographic and Health Surveys (DHS) are established nationally representative household surveys, based on a multi-stage, stratified sampling strategy and designed to provide high-quality information on the health and nutrition of women and children in developing countries [14]. Relevant to this research, the surveys provide linked information on ALRI symptoms among children, household cooking practices and a broad range of other risk factors for child health. We examined DHS surveys conducted in countries of sub-Saharan Africa between 2000 and 2011 for the presence of information on main cooking fuel used; the datasets for 20 countries met this criterion, and the latest surveys were combined in a pooled African dataset. Due to unavailability of key explanatory variables model building for the cooking fuel analysis was undertaken in a pooled dataset of 15 countries (model development dataset), and the final model re-run in a pooled dataset of 18 countries (final dataset; Table 1). As detailed information on cooking practices was only available for selected countries, the cooking location (9 countries) and stove ventilation (6 countries) analyses were undertaken in distinct datasets. The flowchart (Fig 1) illustrates where countries and observations were lost due to missing variables and missing observations. Our study population was defined as children under five years of age; given that several risk factors for ALRI are specific to the household and setting, only *de jure* residents were considered. To avoid non-independent observations and an overrepresentation of households with many children, we focused on the youngest child of every household. All source data are in the public domain and can be downloaded, after registration, from http://www.measuredhs.com.

**Table 1:** Number of observations, cooking fuel and rainy season in final dataset, by country.

Country, year of survey	Final dataset^a^	Clean fuel [%]	Kerosene [%]	Coal or charcoal [%]	Wood [%]	Lower-grade biomass [%]	1^st^ and 2^nd^ rainy season [months]	
**Benin, 2006**	6954	2.5	0.9	17.5	79.1	0.0	5–6	9–12
**Burkina Faso, 2003**	4445	1.3	0.0	3.8	94.7	0.1	6–10	-
**Cameroon, 2004**	1732	9.2	3.4	5.5	81.8	0.0	4–6	-
**Ethiopia, 2005**	2545	0.7	5.1	2.9	86.4	4.9	6–9	2–5
**Ghana, 2008**	1597	7.0	0.3	28.7	63.7	0.3	4–9	-
**Guinea, 2005**	1499	0.1	0.0	99.7	0.3	0.0	6–11	-
**Kenya, 2008–09**	3312	4.7	4.8	21.0	68.1	1.3	4–6	10–11
**Madagascar, 2008–09**	3080	0.4	0.0	23.2	74.4	2.0	11–4	-
**Mali, 2006–07**	6208	0.2	0.0	12.5	85.2	2.2	6–11	-
**Malawi, 2010**	3055	0.8	0.0	7.5	89.3	2.4	11–5	-
**Mozambique, 2003**	4893	1.8	0.4	14.8	82.7	0.3	11–4	-
**Namibia, 2006–07**	2413	28.3	0.2	0.4	70.7	0.4	12–3	-
**Niger, 2006–07**	2004	1.2	0.0	2.3	94.9	1.6	6–10	-
**Senegal, 2005**	1178	23.4	0.0	10.7	63.4	2.5	5–11	-
**Tanzania, 2010**	4263	0.6	1.1	16.9	81.1	0.3	3–5	10–11
**Uganda, 2006–07**	1354	0.1	0.0	13.3	86.3	0.2	9–11	3–5
**Zambia, 2007–08**	3106	11.2	0.0	26.8	61.9	0.1	10–4	-
**Zimbabwe, 2005–06**	2799	24.1	0.0	0.3	75.0	0.6	11–3	-
**Total**	56437	5.1	0.9	15.2	77.9	1.0	-	-

Please note that the South African DHS data from 2003 are not in the public domain and could therefore not be included in this analysis.

^a^ Dataset without missing values in any explanatory variable of the final model (maternal smoking, time to nearest water source, shelter, and vaccination index, paternal and maternal education, child sex and age, birth order, stunting, geographic location).

### Variables

According to a recognised algorithm we identified children with ALRI as those who had ‘cough‘ and ‘short rapid breath‘ or ‘problems in the chest or a blocked or running nose‘ in the two weeks preceding the survey [10–12,15,16].

A published causal diagram of child ALRI determinants [17] distinguishes vulnerability (i.e. nutritional status such as stunting, breastfeeding, birthweight, vaccination, HIV status), exposure to risks (i.e. household air pollution, environmental tobacco smoke, outdoor air pollution, housing, crowding, handwashing), access to health care (i.e. care-seeking, transportation, affordability), household socio-economic status (i.e. maternal and paternal education, maternal and paternal occupation, wealth, income) and contextual factors (i.e. ethnicity, urban/rural location, geographical location). We attempted to populate this diagram with relevant DHS variables at individual/household level (Table 2). In addition, we included child age, sex and birth order as non-modifiable risk factors, and developed the country-level variable rainy season as a further contextual factor to indicate whether the interview took place during the rainy or dry season, with the information obtained from the CIA factbook [18] and other relevant sources (Table 1). All coding of variables was done *a priori* with regard to existing evidence and likely causal mechanisms. Definitions of all variables are provided in Table 2.

**Table 2:** Variables and their population distributions in the model development dataset (15 countries) and final dataset (18 countries), arranged by groups of determinants according to conceptual diagram [17].

Variable	Categories	Model development dataset^a^ (N = 48815) N (%)	Final dataset^b^ (N = 56437) N (%)
Acute Lower Respiratory Infections	Yes	5552 (11.4)	6338 (11.2)
	No	43263 (88.6)	50099 (88.8)
**Non-modifiable risk factors**			
Child sex	Male	24677 (50.6)	28521 (50.5)
	Female	24138 (49.4)	27910 (49.5)
	Missing	-	-
Child age [years]	Median (25th, 75th quantile) [Range]	1 (0, 2) [0, 4]	1 (0, 2) [0, 4]
	Missing	-	-
Birth order	1	8892 (18.2)	10180 (18.0)
	2	8953 (18.3)	10166 (18.0)
	≥3	30970 (63.4)	36091 (63.9)
	Missing	-	-
**Exposure to risks**			
Main cooking fuel	Clean fuels (electricity, LPG, gas, biogas, no food cooked in house)	2477 (5.1)	2856 (5.1)
	Kerosene	480 (1.0)	484 (0.9)
	Coal, charcoal	6732 (13.8)	8570 (15.2)
	Wood	38601 (79.1)	43959 (77.9)
	Lower-grade biomass (straw, shrubs, grass, crop residues, dung)	525 (1.1)	568 (1.0)
Maternal smoking	Yes	3429 (7.0)	3767 (6.7)
	No	45386 (93.0)	52670 (93.3)
	Missing	-	-
Stove ventilation	Cleaner fuels^c^	1154 (2.4)	1182 (2.1)
	Kerosene^c^	10 (0.0)	10 (0.0)
	Wood and lower-grade biomass without stove ventilation	10672 (21.9)	10800 (19.1)
	Wood and lower-grade biomass with stove ventilation	153 (0.3)	153 (0.3)
	Coal, charcoal without stove ventilation	2355 (4.8)	2382 (4.2)
	Coal, charcoal with stove ventilation	33 (0.1)	34 (0.1)
	Not applicable^d^	34395 (70.5)	41832 (74.1)
	Missing	43 (0.1)	44 (0.1)
Cooking location	Cleaner fuels^c^	1993 (4.1)	2032 (3.6)
	Kerosene^c^	298 (0.6)	300 (0.5)
	Solid fuels outdoors	5371 (11.0)	5446 (9.6)
	Solid fuels used in separate building	8718 (17.9)	8800 (15.6)
	Solid fuels used indoors with separate kitchen	3042 (6.2)	3062 (5.4)
	Solid fuels used indoors without separate kitchen	3455 (7.1)	3499 (6.2)
	Not applicable^d^	25817 (52.9)	33176 (58.8)
	Missing	121 (0.2)	122 (0.2)
Crowding	Not crowded (< = 3)	28273 (57.9)	28473 (50.5)
(number of household members per sleeping room)	Crowded (>3)	20542 (42.1)	20670 (36.6)
	Missing	-	7294 (12.9)
Time to nearest water source (minutes)	Median (25th, 75th quantile) [Range]	10 (3, 30) [0, 720]	10 (3, 30) [0, 720]
	Missing	-	-
**Household socio-economic status**			
Shelter index	Low (0–1)	22740 (46.6)	27181 (48.2)
(This index sums over floor, wall and roof material. 0 = natural, 1 = rudimentary, 2 = finished)	Intermediate (2–4)	12265 (25.1)	12411 (22.0)
	High (5–6)	13810 (28.3)	16845 (29.8)
	Missing	-	-
Wealth index	Poorest	10757 (22.0)	12410 (22.0)
(Composite measure of a household’s living standard based on ownership of assets; households are grouped in quintiles)	Poorer	9849 (20.2)	11549 (20.5)
	Middle	9881 (20.2)	11647 (20.6)
	Richer	9604 (19.7)	10869 (19.3)
	Richest	8724 (17.9)	9962 (17.7)
	Missing	-	0 (0.0)
Maternal education	None	20470 (41.9)	26661 (47.2)
	Primary	19030 (39.0)	19965 (35.4)
	Secondary	8579 (17.6)	9042 (16.0)
	Higher	736 (1.5)	769 (1.4)
	Missing	-	-
Paternal education	None	15997 (32.8)	21922 (38.8)
	Primary	17835 (36.5)	18616 (33.0)
	Secondary	10783 (22.1)	11373 (20.2)
	Higher	1685 (3.5)	1840 (3.3)
	Never married^e^	2515 (5.2)	2686 (4.8)
	Missing	-	-
Possession of health card	Yes	41396 (84.8)	47239 (83.7)
	No	7419 (15.2)	9124 (16.2)
	Missing	-	74 (0.1)
**Vulnerability**			
Breastfeeding duration	Currently breastfeeding	28035 (57.4)	32932 (58.4)
	< = 12 month	3112 (6.4)	3239 (5.7)
	12–24 month	14217 (29.1)	15890 (28.2)
	>24 month	2615 (5.4)	3197 (5.7)
	Never	839 (1.7)	910 (1.6)
	Missing	-	269 (0.5)
Stunting (Z-score for height-for-age)	Not stunted (≥-2 SD)	32767 (67.1)	38048 (67.4)
	Stunted (<-2 SD)	16048 (32.9)	18362 (32.5)
	Missing	-	-
Vaccination index	Low (0–3)	11310 (23.2)	13534 (24.0)
(Cumulative vaccine shots against BCG (0,1), DPT (0–3), polio (0–4) and measles (0,1))	Intermediate (4–6)	8662 (17.7)	10152 (18.0)
	High (7–9)	28843 (59.1)	32751 (58.0)
	Missing	-	-
**Contextual factors**			
Rainy season	Yes	26200 (53.7)	31064 (55.0)
	No	22615 (46.3)	25373 (45.0)
	Missing	-	-
Geographic location	Countryside	34700 (71.1)	40660 (72.0)
	Small city	5983 (12.3)	6937 (12.3)
	Town	5111 (10.5)	5445 (9.6)
	Capital, large city	3021 (6.2)	3395 (6.0)
	Missing	-	-
Religion	Christian	25760 (52.8)	27503 (48.7)
	Muslim	12378 (25.4)	17476 (31.0)
	No or other religion	5095 (10.4)	5853 (10.4)
	Unknown religion^f^	5582 (11.4)	5605 (9.9)
Sex of household head	Male	39665 (81.3)	46518 (82.4)
	Female	9150 (18.7)	9919 (17.6)
	Missing	-	-
Age of household head [years]	Median (25th, 75th quantile) [Range]	37 (30, 46) [14, 97]	38 (30, 47) [14, 97]
	Missing	-	28 (0.0)
Number of regions		173	206
Number of countries		15	18

^a^ Dataset without missing values in any explanatory variable (maternal smoking, crowding, time to nearest water source, shelter, wealth, and vaccination index, paternal and maternal education, possession of health card, child sex and age, birth order, breastfeeding duration, stunting, geographic location, sex and age of household head).

^b^ Dataset without missing values in any explanatory variable of the final model (maternal smoking, time to nearest water source, shelter, and vaccination index, paternal and maternal education, child sex and age, birth order, stunting, geographic location).

^c^ Question is not asked if ‘main cooking fuel‘ is a clean fuel or kerosene.

^d^ This question is not asked in all countries.

^e^ This question is not applicable for women who were never married.

^f^ Missing values in religion were combined with category “unknown”.

Cooking variables were defined according to the extent that they are likely to result in exposure to HAP. Fuel type was categorised as clean fuels (i.e. electricity, gas, biogas, no food cooked in house), kerosene, coal and charcoal, wood, and lower-grade biomass fuels (i.e. shrubs, agricultural residues, dung) (Table 1). The skip pattern of the DHS questionnaire, where questions related to cooking location (i.e. indoors vs. outdoors, kitchen located in a separate room or building) and stove ventilation (i.e. presence/absence of a chimney or smoke hood) are only posed to those cooking with solid fuels, made it necessary to combine this information and fuel type in new variables. For the revised cooking location variable, this yielded the categories cleaner fuels, kerosene, solid fuels used outdoors, solid fuels used in separate building, solid fuels used indoors with separate kitchen, and solid fuels used indoors without separate kitchen. The derived stove ventilation variable distinguishes cleaner fuels, kerosene, coal and charcoal with vs. without stove ventilation, and biomass with vs. without stove ventilation (Table 2).

In accordance with published work, we combined the variables floor, wall and roof materials in a shelter index [16,17], and vaccine shots against different diseases in a vaccination index [19]. The wealth index generally used in DHS analyses is a composite measure of a household’s living standard based on ownership of various assets and tends to be used as quintiles [20]. For crowding we followed the United Nations Human Settlements Program (UN Habitat), which defines overcrowding as the presence of more than 3 persons per room [21]. Stunting is based on the height-for-age z-score, with children of <-2 SD classified as stunted according to the WHO cut-off point [22]. Handwashing and environmental tobacco smoke could only be assessed through relatively poor proxies, i.e. time to nearest water source and maternal smoking, respectively. Several latent factors could not be described through variables or proxies (e.g. variables under access to health care, HIV status); the variable birth weight was not included due to poor quality, with mother’s recall of a child’s size at birth being subject to substantial bias [23].

### Statistical analysis

To examine differences in the distribution of variables between the model development dataset and the final dataset we used non-parametric Wilcoxon (two variables) or Kruskal-Wallis tests (more than two variables) for continuous variables, and Chi-squared tests for categorical variables. Correlation between explanatory variables was assessed using Spearman’s rank correlation coefficient. To explore differences in ALRI prevalence for cooking fuel use stratified by rainy and dry season, we used the Mantel-Haenszel test.

We used multi-level mixed models [24] to compare the influence of individual/household-level variables (level 1) and regional (level 2) as well as country-level (level 3) contextual effects on ALRI. Multilevel models separate the variation in an outcome into individual and group- or area-level components [25]; mixed models are especially useful for clustered data, as random effects defined at different levels of the model hierarchy can capture clustering or non-independence of the outcomes [26]. We conducted three separate analyses: The cooking fuel analysis was initially run in the model development dataset to derive a best-fitting model (n_develop_ = 15) and then repeated in the final dataset (n_final_ = 18). Two additional analyses of cooking location and stove ventilation were run in a cooking location (n_location_ = 9) and stove ventilation dataset (n_ventilation_ = 6) respectively (Fig 1).

ALRI was modelled in terms of univariable and multivariable logistic mixed model regressions, including the random variable b_0_ at country level (n_develop_ = 15 countries, n_final_ = 18 countries, n_location_ = 9 countries, n_ventilation_ = 6 countries) and the random variable b_1_ at regional level (n_develop_ = 173 regions, n_final_ = 206 regions, n_location_ = 95 regions, n_ventilation_ = 66 regions), where region refers to the DHS-defined third-level administrative divisions, such as districts or sub-provinces in each country. Thus, the mixed models equation for i = 1,…, n, depending on the number of covariates (X) k, is

$$p_{i}=E\left[Y_{i}|X_{i}\right]=P\left(Y_{i}=1|X_{i}\right)=\frac{\text{exp}\left(X_{i}\beta \right)}{1+\text{exp}\left(X_{i}\beta \right)}$$

with

$$X_{i}\beta =\beta_{0}+\beta_{1}X_{1i}+\cdots +\beta_{k}X_{ki}+b_{0i}Region+b_{1i}Country$$

and

$$Y_{i}|X_{i}∼Ber\left(p_{i}\right)$$

and

$$b_{i}∼MVN\left(0,\Sigma \right).$$

Likelihood-Ratio-Tests (LR-Test) were used to examine the overall effect of variables and interactions in model comparisons.

### Cooking fuel analysis

All variables in Table 2 were considered during model selection. The following interaction terms were also tested: cooking fuel * child age, as the impact of exposure to HAP may depend on age-dependent windows of vulnerability; vaccination index * child age, as vaccinations are administered in an age-dependent fashion; cooking fuel * rainy season, as the type of fuel used and its moisture content are likely to differ between rainy and dry seasons; stunting * child age, as the relevance of stunting for ALRI may vary by age. For model selection, we used a stepwise backward selection algorithm based on the Akaike Information Criterion (AIC) [27], selecting the model with the smallest AIC value as the best-fitting model.

We conducted regional sensitivity analyses to investigate the robustness of effect estimates obtained through the best-fitting model across different regions of sub-Saharan Africa, and to identify any substantial changes in variables that may point to errors in the model. Therefore the best-fitting model was refit on four subsets of the final dataset according to established regional inter-governmental organisations, i.e. the East African Community (EAC, in our analysis Ethiopia, Kenya, Rwanda, Tanzania, and Uganda), the Central African States (CAS; in our analysis Cameroon, Gabon, Ghana, and Guinea), the Southern African Development Community (SADC; in our analysis Madagascar, Malawi, Mozambique, Namibia, Zambia, and Zimbabwe) and the Economic Community of West African States (ECOWAS; in our analysis Benin, Burkina Faso, Mali, Niger, and Senegal).

### Cooking location and stove ventilation analyses

For these analyses, a separate model selection process based on the AIC was undertaken, considering the explanatory variables of interest and all other variables and interactions included in the best-fitting model identified in the cooking fuel analysis.

## Results

The final dataset contained 18 countries and 56,437 observations (Fig 1). Population distributions of outcome and explanatory variables across different stages of information loss are largely comparable (Tables 1 and 2). 6,338 or 11.2% of children were reported to have suffered from ALRI during the two weeks preceding the survey. The proportion of children with ALRI was significantly (p<0.0001) greater during the dry season (13.6%) than during the rainy season (9.3%) (Table 3).

**Table 3:** ALRI frequency, by fuel type and rainy season.

Final dataset^a^		ALRI		
N, (%)		No	Yes	Total
Dry season	Clean fuels	1231 (90.1)	135 (9.9)	1366
	Kerosene	270 (90.0)	30 (10.0)	300
	Coal, charcoal	3961 (88.6)	511 (11.4)	4472
	Wood	16245 (85.4)	2746 (14.6)	18991
	Lower-grade biomass	211 (86.5)	33 (13.5)	244
	Total	21918 (86.4)	3455 (13.6)	25373
Rainy season	Clean fuels	1381 (92.6)	109 (7.4)	1490
	Kerosene	161 (87.5)	23 (12.5)	184
	Coal, charcoal	3675 (89.7)	423 (10.3)	4098
	Wood	22668 (90.8)	2300 (9.2)	24968
	Lower-grade biomass	296 (91.4)	28 (8.6)	324
	Total	28181 (90.7)	2883 (9.3)	31064
Total		50099 (88.8)	6338 (11.2)	56437

^a^ Dataset without missing values in any explanatory variable of the final model (maternal smoking, time to nearest water source, shelter, and vaccination index, paternal and maternal education, child sex and age, birth order, stunting, geographic location).

Cooking fuel use showed much variability across African countries (Table 1). Clean fuel use was least frequent (5.1% of households) whereas use of biomass fuels was very common in all countries, except for Guinea where charcoal use (and to a lesser extent coal use) was most prominent. Stove ventilation through chimneys or smoke hoods was rare (0.4% of households), whereas 25.2% of households cook outdoors or in a separate building (Table 2). It is noteworthy that cooking fuel use differs significantly (p<0.0001) between seasons: During the dry season, households tend to use more coal or charcoal (17.6%) and less wood (74.8%) than during the rainy season (i.e. 13.2% and 80.4% respectively) (Table 3).

### Cooking fuel analysis

Results for the univariable model are shown in Table 4 (left column). The best-fitting model comprised all variables listed in Table 4 (right column), as well as interactions between cooking fuel and season (with a reduced odds of ALRI during the rainy season), as well as interactions between stunting and child age and vaccination index and child age. The variables crowding, possession of health card, breastfeeding duration, sex and age of household head did not improve model fit according to the AIC.

**Table 4:** Results of the logistic mixed model regressions of ALRI with main cooking fuel.

Variables	Categories	Univariable		Multivariable Best-Fitting Models			
		Model development dataset (N = 48815)^a^		Model development dataset (N = 48815)^a^		Final dataset(N = 56437)^b^	
**Exposure to risks**							
Main cooking fuel		OR (95% CI)	p-value	OR (95% CI)	p-value	OR (95% CI)	p-value
	Clean fuels	-	-	-	-	-	-
	Kerosene	1.60 (1.12, 2.28)	0.0099	1.30 (0.81, 2.10)	0.2755	1.23 (0.77, 1.95)	0.3875
	Coal, charcoal	1.68 (1.39, 2.03)	<0.0001	1.46 (1.12, 1.90)	0.0053	1.35 (1.06, 1.72)	0.0139
	Wood	1.65 (1.38, 1.96)	<0.0001	1.38 (1.07, 1.79)	0.0143	1.32 (1.04, 1.66)	0.0209
	Lower-grade biomass	1.51 (1.07, 2.13)	0.0186	1.12 (0.70, 1.79)	0.6429	1.07 (0.69, 1.66)	0.7743
Maternal smoking	Yes	1.20 (1.03, 1.40)	0.0221	1.21 (1.04, 1.42)	0.0147	1.22 (1.06, 1.41)	0.0070
pLR = 0.0080	No	-	-	-	-	-	-
Crowding	Not crowded	-	-	Factor was not selected for the best-fitting model			
	Crowded	1.04 (0.98, 1.10)	0.2048	-			
Time to nearest water source pLR = 0.0013	[coded in 10 min intervals]	1.02 (1.01, 1.03)	<0.0001	1.01 (1.00, 1.02)	<0.0001	1.01 (1.01, 1.02)	0.0010
**Non-modifiable risk factors**							
Child sex	Male	1.10 (1.03, 1.16)	0.0017	1.10 (1.04, 1.17)	<0.0001	1.13 (1.07, 1.19)	<0.0001
pLR < 0.0001	Female	-	-	-	-	-	-
Child age	[years]	0.91 (0.89, 0.94)	<0.0001	0.91 (0.87, 0.94)	<0.0001	0.90 (0.87, 0.93)	<0.0001
Birth order	1						
pLR = 0.0118	2	0.89 (0.81, 0.98)	0.0144	0.88 (0.80, 0.97)	0.0080	0.89 (0.81, 0.97)	0.0104
	≥3	0.90 (0.84, 0.97)	0.0064	0.88 (0.82, 0.96)	0.0023	0.90 (0.83, 0.97)	0.0048
**Household socio-economic status**							
Shelter index pLR = 0.0004	Low	-	-	-	-	-	-
	Intermediate	0.99 (0.91, 1.07)	0.8175	1.00 (0.93, 1.09)	0.9257	1.02 (0.94, 1.10)	0.6996
	High	0.78 (0.72, 0.85)	<0.0001	0.83 (0.75, 0.92)	0.0002	0.86 (0.79, 0.94)	<0.0001
Wealth index	Poorest	-	-	Factor was not selected for the best-fitting model instead shelter index was selected			
	Poorer	0.98 (0.90, 1.07)	0.6410	-			
	Middle	0.96 (0.87, 1.04)	0.3156	-			
	Richer	0.87 (0.79, 0.95)	0.0033	-			
	Richest	0.75 (0.68, 0.84)	<0.0001	-			
Maternal education	None	1.30 (1.00, 1.70)	0.0533	0.87 (0.64, 1.17)	0.3474	0.91 (0.68, 1.22)	0.5265
pLR = 0.0002	Primary	1.46 (1.13, 1.90)	0.0043	1.02 (0.77, 1.37)	0.8693	1.08 (0.82, 1.44)	0.5782
	Secondary	1.26 (0.97, 1.64)	0.0880	0.96 (0.72, 1.27)	0.7530	1.01 (0.77, 1.34)	0.9313
	Higher	-	-	-	-	-	-
Paternal education	None	1.53 (1.27, 1.84)	<0.0001	1.36 (1.11, 1.68)	0.0037	1.29 (1.06, 1.57)	0.0102
pLR = 0.1148	Primary	1.44 (1.20, 1.72)	<0.0001	1.23 (1.01, 1.50)	0.0400	1.22 (1.01, 1.48)	0.0363
	Secondary	1.38 (1.15, 1.65)	<0.0001	1.23 (1.02, 1.50)	0.0346	1.20 (1.00, 1.45)	0.0481
	Higher	**-**	-	-	-	-	-
	Never married^d^	1.41 (1.14, 1.75)	0.0018	1.15 (0.92, 1.46)	0.2250	1.16 (0.93, 1.45)	0.1833
Possession of health card	Yes	0.99 (0.91, 1.08)	0.7887	Factor was not selected for the best-fitting model			
	No	-	-	-			
**Vulnerability**							
Breastfeeding duration	Currently breastfeeding	1.23 (0.97, 1.55)	0.0891	Factor was not selected for the best-fitting model			
	<12 month	1.04 (0.80, 1.35)	0.7492	-			
	12–24 month	1.01 (0.80, 1.29)	0.9092	-			
	>24 month	1.10 (0.84, 1.43)	0.4845	-			
	Never	-	-	-			
Stunting	Not stunted	-	-	-	-	-	-
	Stunted	1.02 (0.96, 1.09)	0.4513	1.12 (1.01, 1.23)	0.0256	1.11 (1.01, 1.22)	0.0290
Vaccination index	Low	0.97 (0.90, 1.05)	0.4787	0.83 (0.75, 0.92)	<0.0001	0.85 (0.77, 0.93)	<0.0001
	Intermediate	1.10 (1.02, 1.19)	0.0181	0.97 (0.87, 1.07)	0.5179	0.98 (0.89, 1.09)	0.7296
	High	-	-	-	-	-	-
**Contextual factors**							
Rainy season	Yes	0.74 (0.68, 0.80)	<0.0001	0.76 (0.55, 1.04)	0.0907	0.79 (0.59, 1.05)	0.1079
	No	-	-	-	-	-	-
Geographic location	Countryside	1.06 (0.85, 1.32)	0.6137	0.90 (0.71, 1.14)	0.3941	0.87 (0.70, 1.09)	0.2228
pLR = 0.0212	Small city	0.89 (0.70, 1.14)	0.3529	0.79 (0.62, 1.01)	0.0625	0.75 (0.60, 0.95)	0.0147
	Town	0.92 (0.72, 1.16)	0.4673	0.88 (0.69, 1.13)	0.3158	0.86 (0.68, 1.07)	0.1717
	Capital, large city	-	-	-	-	-	-
Religion	Christian	-	-	-	-	-	-
pLR = 0.0088	Muslim	0.91 (0.81, 1.02)	0.0908	0.89 (0.80, 1.00)	0.0549	0.88 (0.80, 0.97)	0.0132
	No or other religion	1.03 (0.93, 1.15)	0.5420	0.99 (0.88, 1.10)	0.8088	0.98 (0.88, 1.09)	0.7190
	Unknown religion	0.78 (0.64, 0.95)	0.0147	0.76 (0.62, 0.92)	0.0060	0.78 (0.64, 0.95)	0.0131
Sex of household head	Male	0.97 (0.90, 1.04)	0.3472	Factor was not selected for the best-fitting model			
	Female	-	-	-			
Age of household head	[years]	1.00 (1.00, 1.00)	0.1424	Factor was not selected for the best-fitting model			
**Interactions**							
Main cooking fuel * rainy season	Clean fuels	-	-	-	-	-	-
^c^pLR<0.0001, <0.0341	Kerosene	**-**	-	1.33 (0.67, 2.63)	0.4111	1.34 (0.69, 2.59)	0.3884
	Coal, charcoal	**-**	-	1.16 (0.81, 1.65)	0.4149	1.14 (0.83, 1.58)	0.4129
	Wood	**-**	-	0.94 (0.68, 1.30)	0.7142	0.91 (0.68, 1.23)	0.5443
	Lower-grade biomass	**-**	-	1.30 (0.67, 2.54)	0.4373	1.39 (0.75, 2.57)	0.3008
Stunting * child age	Not stunted	-	-	-	-	-	-
^c^pLR = 0.0792, 0.0411	Stunted	**-**	-	0.94 (0.89, 0.99)	0.0300	0.95 (0.90, 1.00)	0.0413
Vaccination index * child age	Low	-	-	1.06 (0.99, 1.12)	0.0850	1.05 (0.99, 1.11)	0.1118
^c^pLR< 0.0014, 0.1495	Intermediate	-	-	1.06 (0.99, 1.13)	0.0761	1.05 (0.99, 1.11)	0.1259
	High	-	**-**	**-**	-	-	-
Main cooking fuel * child age		-	**-**	Factor was not selected for the best-fitting model			

* refers to an interaction between two variables. Please note, the presented ORs are coming from the estimate of the corresponding beta and are of pure statistical interest. Interpretable ORs for variables with interaction terms can be found in the text.

pLR is the p-value of the LR-Test comparing the final model with the final model without the corresponding covariate.

^a^ Dataset without missing values in any explanatory variable (maternal smoking, crowding, time to nearest water source, shelter, wealth, and vaccination index, paternal and maternal education, possession of health card, child sex and age, birth order, breastfeeding duration, stunting, geographic location, sex and age of household head).

^b^ Dataset without missing values in any explanatory variable of the final model (maternal smoking, time to nearest water source, shelter, and vaccination index, paternal and maternal education, child sex and age, birth order, stunting, geographic location).

^c^ Both p-values relate to LR-Tests comparing (i) the final model with a model without main or interaction effects, and (ii) the final model with a model with main effects but without interaction effects.

^d^ Women who were never married are per design of the questionnaires not asked about the education of their husband/partner.

In the best-fitting multivariable model for the final dataset (Table 4, right column; see S1 Appendix for derivation of ORs including interactions), cooking fuel was associated with statistically significant differences in ALRI (p<0.0001). During the rainy season, relative to clean fuels, the odds of suffering from ALRI were raised for kerosene (OR 1.64; CI: 0.99, 2.71), coal and charcoal (OR 1.54; CI: 1.21, 1.97), wood (OR 1.20; CI: 0.95, 1.51) and lower-grade biomass fuels (OR 1.49; CI: 0.93, 2.35). In contrast, during the dry season the corresponding odds were reduced for kerosene (OR 1.23; CI: 0.77, 1.95), coal and charcoal (OR 1.35; CI: 1.06, 1.72) and lower-grade biomass fuels (OR 1.07; CI: 0.69, 1.66) but increased for wood (OR 1.32; CI: 1.04, 1.66).

Maternal smoking (OR 1.22, CI: 1.06, 1.41), living far from the nearest water source (OR 1.01; CI: 1.01, 1.02 for an increased travelling time of ten minutes), being a male (OR 1.13; CI: 1.07, 1.19) or first-born child (second-born child: OR: 0.89; CI: 0.81, 0.97; all later-born children: OR 0.90; CI: 0.83, 0.97), being stunted (greater effect for younger compared to older children) and having a higher vaccination index (greater effect for younger compared to older children) all increased the odds of ALRI with statistical significance. On the other hand, a higher shelter index (OR 0.86, CI: 0.79, 0.94 for high compared to low shelter index), greater parental education (maternal and paternal should be interpreted jointly, as they are highly correlated), being Muslim (OR 0.88; CI: 0.80, 0.97 compared to being Christian) and living in a small city (OR 0.75; CI: 0.60, 0.95 compared to living in the capital or in a large city) acted as statistically significant protective factors against ALRI.

The four regional sensitivity analyses showed no substantial changes in effect estimates (S2 Table).

### Cooking location and stove ventilation analyses

In the univariable models, cooking location (p<0.0001) and stove ventilation (p = 0.0045) both increased ALRI risk (S1 Table). Cooking location also showed substantial differences between seasons (p<0.0001): During the rainy season, cooking in a separate building is substituted (28.3% compared to 45.0% during dry season) with cooking indoors in a separate kitchen (20.2% compared to 8.2% during dry season) or main room (17.4% compared to 13.5% during dry season) (see S1 Appendix for derivation of ORs including interactions). While the stove ventilation variable also showed statistically significant seasonal differences (p<0.0001), these were attributable to a greater use of clean fuels during the rainy season rather than to a greater or lesser use of chimneys or smoke hoods.

In the best-fitting multivariable model cooking location emerged as a statistically significant (p = 0.0002) risk factor for ALRI, and significantly (p = 0.0160) interacted with rainy season (Table 5). During the rainy season, relative to cooking with clean fuels the odds of ALRI were increased for use of solid fuels outdoors (OR 1.70, CI 1.25, 2.31), in a separate building (OR 1.58; CI: 1.16, 2.14), indoors in a separate kitchen (OR 1.22, CI: 0.87, 1.71) and indoors with no separate kitchen (OR 1.80, CI: 1.30, 2.50). During the dry season, the corresponding odds of suffering from ALRI were as follows: cooking outdoors (OR 1.36; CI: 1.00, 1.86), cooking in a separate building (OR 1.55; CI: 1.14, 2.11), cooking indoors with separate kitchen (OR 1.65; CI: 1.18, 2.32) and cooking indoors with no separate kitchen (OR 1.51; CI: 1.09, 2.10). In this analysis, cooking with kerosene increased the odds of ALRI more during the rainy season (OR 2.50, CI: 1.33, 4.73) than during the dry season (OR 1.56, CI: 0.87, 2.83). These estimates are adjusted for time to nearest water source, shelter index, maternal education, birth order, stunting, child age, child sex and an interaction term for stunting and child age.

**Table 5:** Results of the logistic mixed model regressions of ALRI for stove ventilation and cooking location.

Variables	Categories	Best-Fitting Multivariable Stove Ventilation Model N = 14561, 6 countries^a^		Best-Fitting Multivariable Cooking Location Model N = 23139, 9 countries^b^	
		OR (95% CI)	p-value	OR (95% CI)	p-value
**Exposure to risks**					
Stove ventilation	Clean fuels	-	-	Not applicable	
pLR = 0.1280	Kerosene	nc.	nc.	Not applicable	
	Coal without stove ventilation	1.44 (1.08, 1.92)	0.0136	Not applicable	
	Coal with stove ventilation	1.66 (0.60, 4.58)	0.3278	Not applicable	
	Biomass fuels without stove ventilation	1.42 (1.07, 1.88)	0.0137	Not applicable	
	Biomass fuels with stove ventilation	1.36 (0.80, 2.31)	0.2627	Not applicable	
Cooking location	Clean fuels	Not applicable		-	-
	Kerosene	Not applicable		1.56 (0.87, 2.83)	0.1386
	Solid fuels outdoors	Not applicable		1.36 (1.00, 1.86)	0.0505
	Solid fuels separate building	Not applicable		1.55 (1.14, 2.11)	0.0049
	Solid fuels indoors, separate kitchen	Not applicable		1.65 (1.18, 2.32)	0.0036
	Solid fuels indoors, no separate kitchen	Not applicable		1.51 (1.09, 2.10)	0.0141
Maternal smoking		Factor was not selected for the best-fitting model		Factor was not selected for the best-fitting model	
Time to nearest water source	[coded in 10 min intervals]	1.02 (1.00, 1.03)	0.0476	1.01 (1.00, 1.02)	0.0143
**Non-modifiable risk factors**					
Child sex	Male	1.10 (0.99, 1.22)	0.0708	1.08 (1.00, 1.17)	0.0455
	Female	-	-	-	-
Child age	[years]	0.95 (0.91, 1.00)	0.0646	0.95 (0.91, 0.98)	0.0059
Birth order	1	-	-	-	-
	2	0.90 (0.76, 1.06)	0.2032	0.93 (0.82, 1.05)	0.2315
	≥3	0.82 (0.72, 0.94)	0.0039	0.88 (0.79, 0.98)	0.0179
**Household socio-economic status**					
Shelter index	Low	-	-	-	-
	Intermediate	1.05 (0.92, 1.19)	0.4953	0.98 (0.89, 1.09)	0.7079
	High	0.83 (0.69, 0.99)	0.0394	0.76 (0.66, 0.86)	<0.0001
Maternal education	None	Factor was not selected for the best-fitting model		1.02 (0.74, 1.40)	0.9171
	Primary	-		1.14 (0.84, 1.54)	0.4136
	Secondary	-		1.00 (0.74, 1.36)	0.9812
	Higher	-		-	-
Paternal education		Factor was not selected for the best-fitting model		Factor was not selected for the best-fitting model	
**Vulnerability**					
Stunting	Not stunted	-	-	-	-
	Stunted	1.31 (1.10, 1.56)	0.0020	1.19 (1.04, 1.37)	0.0111
Vaccination index		Factor was not selected for the best-fitting model		Factor was not selected for the best-fitting model	
**Contextual factors**					
Rainy season	Yes	Factor was not selected for the best-fitting model		1.02 (0.70, 1.47)	0.9903
	No	-		-	-
Geographic location		Factor was not selected for the best-fitting model		Factor was not selected for the best-fitting model	
Religion		Factor was not selected for the best-fitting model		Factor was not selected for the best-fitting model	
**Interactions**					
Stove ventilation * rainy season		Factor was not selected for the best-fitting model		Not applicable	
Cooking location* rainy season	Clean fuels	Not applicable		-	-
^c^pLR = 0.0002, 0.0160	Kerosene	Not applicable		1.60 (0.70, 3.65)	0.2636
	Solid fuels outdoors	Not applicable		1.25 (0.83, 1.87)	0.2873
	Solid fuels separate building	Not applicable		1.02 (0.69, 1.51)	0.9311
	Solid fuels indoors, separate kitchen	Not applicable		0.74 (0.47, 1.15)	0.1833
	Solid fuels indoors, no separate kitchen	Not applicable		1.19 (0.78, 1.82)	0.4152
Stunting * child age	Not stunted	-	-	-	-
	Stunted	0.88 (0.80, 0.97)	0.0086	0.91 (0.85, 0.98)	0.01031
Vaccination index * child age		Factor was not selected for the best-fitting model		Factor was not selected for the best-fitting model	
Main cooking fuel * child age		Factor was not selected for the best-fitting model		Factor was not selected for the best-fitting model	

* refers to an interaction between two variables. Please note, the presented ORs are coming from the estimate of the corresponding beta and are of pure statistical interest. Interpretable ORs for variables with interaction terms can be found in the text.

pLR is the p-value of the LR-Test comparing the final model with the final model without the corresponding covariate.

nc. Not calculable due to very low number of households cooking with kerosene (n = 10 housholds were coded as “Clean fuel”).

^a^ Dataset without missing values in any explanatory variable of the final model (maternal smoking, time to nearest water source, shelter, and vaccination index, paternal and maternal education, child sex and age, birth order, stunting, geographic location) for the following countries: Ghana, Madagascar, Malawi, Namibia, Uganda, and Zambia

^b^ Dataset without missing values in any explanatory variable of the final model (maternal smoking, time to nearest water source, shelter, and vaccination index, paternal and maternal education, child sex and age, birth order, stunting, geographic location) for the following countries: Ethiopia, Ghana, Kenya, Madagascar, Malawi, Namibia, Uganda, Zambia, and Zimbabwe

^c^ Both p-values relate to LR-Tests comparing (i) the final model with a model without main or interaction effects, and (ii) the final model with a model with main effects but without interaction effects.

In contrast, stove ventilation was not selected in the best-fitting model, and did not show an independent effect on ALRI (p = 0.1280) (Table 5).

## Discussion

### Key findings

This analysis clearly demonstrated, for the first time in a large pooled routinely collected dataset, the impact of cooking practices on child ALRI. It goes beyond previous analyses in clearly showing differential impacts for different cooking fuel types and in identifying season as a critical factor. While the relationship between temperature, humidity and ALRI incidence is common knowledge [28, 29, 30] and differences in cooking practices between dry and rainy seasons have been described, the interactions between these factors have not been documented at such a large scale across multiple countries. Interestingly, the effect estimates for all fuel types (apart from wood, where findings were largely similar between seasons) were lower during the dry season.

This pooled analyses also provided evidence for cooking location as an important determinant of child ALRI risk, in particular during the rainy season. During the dry season, outdoor cooking (OR 1.36; CI: 1.00, 1.86), was, after cooking with clean fuels, the healthiest option with all other cooking location alternatives increasing the odds of ALRI to a similar extent. During the rainy season, having a separate kitchen indoors emerged, after cooking with clean fuels, as the healthiest option (OR 1.22; CI: 0.87, 1.71); the other cooking locations increased the odds of ALRI with statistical significance, cooking indoors with no separate kitchen to the greatest extent (OR 1.80; CI: 1.30, 2.50). The analysis could, however, not demonstrate an effect of the presence of chimneys or smoke hoods.

Noteworthy are the findings for kerosene, until recently considered a clean fuel, whose use appears to constitute a risk for ALRI comparable in magnitude to that of the use of wood or charcoal and coal in the main cooking fuel analysis; at least for the rainy season, the effect estimate is of borderline statistical significance (OR 1.64; CI: 0.99, 2.71). Surprisingly, with ORs of 1.56 (CI: 0.87, 2.83) and 2.50 (CI: 1.33, 4.73) during the dry and rainy seasons respectively, it shows an even more pronounced effect in the smaller dataset used to assess the impact of cooking fuel and, at least in this analysis, is a more significant risk factor for child ALRI than solid fuel use.

### Explaining and locating findings in the literature

Our effect estimates for the impact of solid fuel use on ALRI lie below those found in the recent systematic review by Dherani and colleagues [3] (OR 1.78; CI: 1.45, 2.18). However, our observed effect sizes during the rainy season were similar to the results obtained when pooling all three previously published cross-sectional studies (OR 1.49; CI: 1.21, 1.85) [3]. As further discussed below, child respiratory symptoms as reported by the mother are a poor measure of ALRI. Likewise, main cooking fuel is an imprecise proxy for HAP. This is due to a combination of factors, including the fuel stacking phenomenon, i.e. the parallel use of multiple fuels (in particular for clean fuel users) [31,32] for cooking, heating and other household energy purposes, and user behaviour in relation to fuel preparation and stove operation. Overall, both of these measurement imprecisions are likely to bias findings towards the null.

The effect size for solid fuels across different cooking locations is largely in line with previous research [3,31,32]. On the other hand, our lack of findings for stove ventilation contradicts the growing evidence base for the effectiveness of chimneys and other types of stove ventilation as a means of reducing HAP [15,32,33,34] and improving respiratory health [35,36,37]. It is probably largely attributable to chimneys or smoke hoods not being widely used in the six countries, for which the analysis could be conducted. Indeed, only 35 coal and charcoal users and 153 biomass users out of a total of 14,561 total respondents cooked on a ventilated stove.

The relationship between season and ALRI incidence is well established, with lower temperatures and greater humidity (both coinciding during the rainy season) being protective against ALRI [28,29,30]. However, this knowledge appears to be insufficiently used in HAP studies, especially in view of the observed interactions between season and fuel type. While there is much overlap in the confidence intervals for the effect estimates for the different fuels by season, the differences in the point estimates relative to the size of the effect are substantial, and lend support to the observed effect modification being real. A partial explanation for the latter is the fact that, during the rainy season, coal and charcoal is replaced with wood, probably due to limited availability or accessibility of cleaner fuels due to a poor supply or transportation infrastructure. A further explanation are changes in behaviour with more time spent cooking indoors during the rainy season. Season therefore influences effect estimates for the impact of cooking practices on child ALRI, in particular, if studies conducted at different times of year are pooled in meta-analyses or, as in our study, if the population within a given study is spread over different climatic zones and/or measurements take place at different times of year.

### Strengths and limitations

One of the biggest challenges in assessing child acute respiratory infections is the distinction between harmless AURI, such as the common cough or cold, and potentially life-threatening ALRI, such as pneumonia and bronchiolitis, which account for a minority of approximately 10% of all acute respiratory infections. Most accurately, a physician's diagnosis of pneumonia is confirmed by an X-ray of the lungs [38]. While field staff can be trained to recognise key symptoms of pneumonia, such assessments are relatively non-specific. Maternal reporting is even less reliable and, beyond problems with the correct recognition of symptoms, may be subject to differential reporting (e.g. by educational status, for male and female children) and recall bias. Consequently, questions have been raised whether the DHS are a suitable data source for examining effects of HAP on child ALRI and whether selective publishing of DHS-based analyses may contribute to the publication bias observed in the systematic review by Dherani and colleagues [3].

In our pooled African dataset we found an ALRI prevalence of 11%. Interestingly, our estimated prevalence is much lower than that reported in three other DHS-based analyses, which observed 16% for Zimbabwe [10], 19% for South Africa [11] and 20% for India [12]; it is also clearly below the low-prevalence threshold of 15% defined by Dherani and colleagues [3]. This observation lends support to the concern about the non-specific assessment of ALRI in country-based DHS analyses and selective publishing of positive findings. On the other hand, the fact that we were able to document consistent effects of cooking practices across a broad range of countries implies that sufficiently large sample sizes can partially overcome this problem. Overall, with AURI misclassified as ALRI, effect estimates based on individual or pooled cross-sectional surveys will be biased towards the null.

Longitudinal studies are generally preferred to cross-sectional studies to answer complex public health issues. Interestingly, recent research by Markovitz [39] showed that cross-sectional studies can yield more precise effect estimates, if factors vary more across space than time. DHS datasets are characterised by good data quality as a result of standardised approaches to sampling, data collection and data entry, which have benefited from improvements over time, and high response rates in each country (between 88% and 99% for the countries in our final dataset). Our analysis is, however, hampered by the large number of missing observations (approximately one quarter was caused by missing variables and three quarters by missing values, see Fig 1) in all three analyses, i.e. 50.3% in the cooking fuel, 53.4% in the cooking location and 59.1% in the stove ventilation analysis. As neither the comparison of the dataset without missing observations and the final dataset nor the regional sensitivity analyses provided any evidence of bias and as the absolute number of complete observations was still very large, we did not use imputation techniques. Nevertheless, as part of a methodological research project, we did repeat the analysis with imputation of major explanatory variables, and the effect estimates turned out to be very stable (Max Siebold and Hannes Buchner, forthcoming publication). Survivor bias may be present in our analysis, as we focused on children who were still alive at the time of data collection. Moreover, we cannot exclude the possibility of temporal changes in measured or unmeasured factors over a time period of more than a decade, as represented in our pooled dataset.

One of the major strengths of the present analysis is that it is embedded in an *a priori* system-based approach to research conception and data analysis, which tries to put into practice the concept of systems epidemiology [40]. Confounders and competing risk factors were addressed comprehensively by drawing on a published causal diagram of ALRI determinants [17] and complemented with explicit considerations of the influence of season on both risk factors and disease. The combination of epidemiological knowledge and statistical techniques in dealing with complex systems identified a large number of relevant and statistically significant variables.

In summary, the present research has shown that sufficiently large DHS datasets can be suitable for studying the effects of cooking practices on child ALRI, provided confounding and competing risk factors are handled in an appropriate way. While our final sample of 18 countries was not designed to be representative of the whole of Africa, it does include a broad range of different geographical and climatic areas across South, East and West Africa and the findings are therefore likely to be applicable across many different parts of the continent.

## Conclusions

This pooled analysis of routinely collected nationally representative data for 18 African countries clearly shows an elevated risk of ALRI for children living in households cooking with solid fuels and children living in households cooking with kerosene. It demonstrates that DHS data can be suitable for studying the impact of household air pollution on health but also confirms previous concerns with respect to imprecise health outcome and exposure measurements. In addition, this analysis has identified cooking location, in particular cooking indoors without a separate kitchen, as an additional determinant of ALRI risk and has highlighted season as a risk factor for ALRI and an effect modifier for the risks associated with cooking. Future primary studies, as well as meta-analyses, should therefore examine potential effect modifications of the relation between household air pollution and ALRI by season. In addition, risks do not only vary over time but also across districts or sub-provinces. These considerations must be taken into account in the planning and conduct of future observational and experimental studies.

## Supporting Information

S1 Appendix A Derivation of ORs including interactions. (DOCX)

S1 Table Univariable results of the logistic mixed model regressions of ALRI for stove ventilation and cooking location. nc. Not calculable due to very low number of households cooking with kerosene (n = 10 housholds were coded as “Clean fuel”). pLR is the p-value of the LR-Test comparing the model without fixed effects with the model with the corresponding covariate. ^a^ Dataset without missing values in any explanatory variable of the final model (maternal smoking, time to nearest water source, shelter, and vaccination index, paternal and maternal education, child sex and age, birth order, stunting, geographic location) for the following countries: Ghana, Madagascar, Malawi, Namibia, Uganda, and Zambia. ^b^ Dataset without missing values in any explanatory variable of the final model (maternal smoking, time to nearest water source, shelter, and vaccination index, paternal and maternal education, child sex and age, birth order, stunting, geographic location) for the following countries: Ethiopia, Ghana, Kenya, Madagascar, Malawi, Namibia, Uganda, Zambia, and Zimbabwe.
(DOCX)

S2 Table Results of the multivariable logistic mixed model regressions of ALRI with main cooking fuel by regional groups. nc. Not calculable due to very low number of households with this characteristic in this region (unknown religion / Lower-grade biomass fuel). * refers to an interaction between two variables. Please note, the presented ORs are coming from the estimate of the corresponding beta. ^a^ Dataset without missing values in any explanatory variable of the final model (maternal smoking, time to nearest water source, shelter, and vaccination index, paternal and maternal education, child sex and age, birth order, stunting, geographic location) for Ethiopia, Kenya, Tanzania, and Uganda. ^b^ Dataset without missing values in any explanatory variable of the final model (maternal smoking, time to nearest water source, shelter, and vaccination index, paternal and maternal education, child sex and age, birth order, stunting, geographic location) for Cameroon, Ghana, and Guinea. ^c^ Dataset without missing values in any explanatory variable of the final model (maternal smoking, time to nearest water source, shelter, and vaccination index, paternal and maternal education, child sex and age, birth order, stunting, geographic location) for Madagascar, Malawi, Mozambique, Namibia, Zambia, and Zimbabwe. ^d^ Dataset without missing values in any explanatory variable of the final model (maternal smoking, time to nearest water source, shelter, and vaccination index, paternal and maternal education, child sex and age, birth order, stunting, geographic location) for Benin, Burkina Faso, Mali, Niger, and Senegal. ^e^ Women who were never married are per design of the questionnaires not asked about the education of their husband/partner.
(DOCX)
