# Supplementary material for: Cooking and Season as Risk Factors for Acute Lower Respiratory Infections in African Children: A Cross-Sectional Multi-Country Analysis
Source: PLoS One. 2015 Jun 4;10(6):e0128933. doi: 10.1371/journal.pone.0128933 (PMC4456387; doi:10.1371/journal.pone.0128933)
Supplement: S1 Appendix — (DOCX) [file pone.0128933.s001.docx]

Odds are defined as $p_{i}/(1-p_{i})=exp(X_{i}\beta$). ORs can be calculated by dividing Odds for different covariates e.g.

$$\mathrm{OR}\left( season=rainy, fuel=wood vs. season=rainy, fuel=clean \right)=$$

$$\frac{\mathrm{Odds}\left( season=rainy, fuel=wood \right)}{\mathrm{Odds}\left( season=rainy, fuel=clean \right)}=$$

$$\frac{\exp\left( \beta_{\mathrm{rainy}} + \beta_{\mathrm{wood}} +\beta_{\mathrm{rainy}*\mathrm{wood}} + Z\beta\right)}{\exp\left( \beta_{\mathrm{rainy}} + Z\beta\right)}$$

because clean fuel is the reference category. This yields

$$\exp\left( \beta_{\mathrm{wood}} +\beta_{\mathrm{rainy}*\mathrm{wood}} \right).$$

Since Table 4 and 5 show $\exp\left( \beta\right)$ one must insert the logarithm of the values presented in these tables:

$$\exp\left( ln(1.32)+ln(0.91) \right)=1.20$$

CIs of effects including interactions are calculated by re-estimating the model with different reference categories of the involved covariates (e.g. instead of defining the dry season as a reference category [original model] the rainy season is now used as the reference category).
