# Supplementary material for: Cooking and Season as Risk Factors for Acute Lower Respiratory Infections in African Children: A Cross-Sectional Multi-Country Analysis
Source: PLoS One. 2015 Jun 4;10(6):e0128933. doi: 10.1371/journal.pone.0128933 (PMC4456387; doi:10.1371/journal.pone.0128933)
Supplement: S1 Table — nc. Not calculable due to very low number of households cooking with kerosene (n = 10 housholds were coded as “Clean fuel”). pLR is the p-value of the LR-Test comparing the model without fixed effects with the model with the corresponding covariate. a Dataset without missing values in any explanatory variable of the final model (maternal smoking, time to nearest water source, shelter, and vaccination index, paternal and maternal education, child sex and age, birth order, stunting, geographic location) for the following countries: Ghana, Madagascar, Malawi, Namibia, Uganda, and Zambia. b Dataset without missing values in any explanatory variable of the final model (maternal smoking, time to nearest water source, shelter, and vaccination index, paternal and maternal education, child sex and age, birth order, stunting, geographic location) for the following countries: Ethiopia, Ghana, Kenya, Madagascar, Malawi, Namibia, Uganda, Zambia, and Zimbabwe. (DOCX) [file pone.0128933.s002.docx]

| **Variables** | **Categories** | **Stove Ventilation N=14561, 6 countries^a^** | | **Cooking Location N=23139, 9 countries^b^** | |
| --- | --- | --- | --- | --- | --- |
|  |  | OR (95% CI) | p-value | OR (95% CI) | p-value |
| **Exposure to risks** | | | | | |
| Stove ventilation | Clean fuels | - | - | Not applicable | |
| pLR=0.0045 | Kerosene | nc. | nc. | Not applicable | |
|  | Coal without stove ventilation | 1.50 (1.13, 2.00) | 0.0051 | Not applicable | |
|  | Coal with stove ventilation | 1.78 (0.65, 4.92) | 0.2637 | Not applicable | |
|  | Biomass fuels without stove ventilation | 1.63 (1.26, 2.11) | 0.0002 | Not applicable | |
|  | Biomass fuels with stove ventilation | 1.52 (0.90, 2.56) | 0.1169 | Not applicable | |
| Cooking location | Clean fuels | Not applicable | | - | - |
| pLR<0.0001 | Kerosene | Not applicable | | 2.01 (1.28, 3.16) | 0.0023 |
|  | Solid fuels outdoors | Not applicable | | 1.82 (1.47, 2.26) | <0.0001 |
|  | Solid fuels separate building | Not applicable | | 1.96 (1.59, 2.43) | <0.0001 |
|  | Solid fuels indoors, separate kitchen | Not applicable | | 1.74 (1.37, 2.21) | <0.0001 |
|  | Solid fuels indoors, no separate kitchen | Not applicable | | 2.07 (1.64, 2.62) | <0.0001 |
| Time to nearest water source | [coded in 10 min intervals] | 1.02 (1.00, 1.03) | 0.0115 | 1.02 (1.01, 1.03) | <0.0001 |
| **Non-modifiable risk factors** | | | | | |
| Child sex | Male | 1.10 (0.99, 1.21) | 0.0769 | 1.08 (1.00, 1.17) | 0.0560 |
|  | Female | - | - | - | - |
| Child age | [years] | 0.92 (0.88, 0.96) | <0.0001 | 0.92 (0.89, 0.95) | <0.0001 |
| Birth order | 1 | - | - | - | - |
|  | 2 | 1.19 (1.04, 1.35) | 0.0117 | 0.94 (0.83, 1.06) | 0.3109 |
|  | ≥3 | 1.07 (0.94, 1.23) | 0.2967 | 0.92 (0.83, 1.02) | 0.1258 |
| **Household socio-economic status** | | | | | |
| Shelter index | Low | - | - | - | - |
|  | Intermediate | 1.03 (0.90, 1.17) | 0.6994 | 0.97 (0.88, 1.07) | 0.5428 |
|  | High | 0.77 (0.66, 0.90) | 0.0013 | 0.68 (0.60, 0.77) | <0.0001 |
| Maternal education | None | Not applicable | | 1.44 (1.06, 1.94) | 0.0185 |
|  | Primary | Not applicable | | 1.57 (1.18, 2.10) | 0.0021 |
|  | Secondary | Not applicable | | 1.27 (0.95, 1.70) | 0.1121 |
|  | Higher | Not applicable | | - | - |
| **Vulnerability** | | | | | |
| Stunting | Not stunted | - | - | - | - |
|  | Stunted | 1.08 (0.97, 1.21) | 0.1445 | 1.04 (0.95, 1.13) | 0.4125 |
| **Contextual factors** | | | | | |
| Rainy season | Yes | Not applicable | | 1.03 (0.93, 1.14) | 0.5757 |
|  | No | Not applicable | | - | - |
