# Supplementary material for: Cooking and Season as Risk Factors for Acute Lower Respiratory Infections in African Children: A Cross-Sectional Multi-Country Analysis
Source: PLoS One. 2015 Jun 4;10(6):e0128933. doi: 10.1371/journal.pone.0128933 (PMC4456387; doi:10.1371/journal.pone.0128933)
Supplement: S2 Table — nc. Not calculable due to very low number of households with this characteristic in this region (unknown religion / Lower-grade biomass fuel). * refers to an interaction between two variables. Please note, the presented ORs are coming from the estimate of the corresponding beta. a Dataset without missing values in any explanatory variable of the final model (maternal smoking, time to nearest water source, shelter, and vaccination index, paternal and maternal education, child sex and age, birth order, stunting, geographic location) for Ethiopia, Kenya, Tanzania, and Uganda. b Dataset without missing values in any explanatory variable of the final model (maternal smoking, time to nearest water source, shelter, and vaccination index, paternal and maternal education, child sex and age, birth order, stunting, geographic location) for Cameroon, Ghana, and Guinea. c Dataset without missing values in any explanatory variable of the final model (maternal smoking, time to nearest water source, shelter, and vaccination index, paternal and maternal education, child sex and age, birth order, stunting, geographic location) for Madagascar, Malawi, Mozambique, Namibia, Zambia, and Zimbabwe. d Dataset without missing values in any explanatory variable of the final model (maternal smoking, time to nearest water source, shelter, and vaccination index, paternal and maternal education, child sex and age, birth order, stunting, geographic location) for Benin, Burkina Faso, Mali, Niger, and Senegal. e Women who were never married are per design of the questionnaires not asked about the education of their husband/partner. (DOCX) [file pone.0128933.s003.docx]

| **Variables** | **Categories** | **EAC N=11474, 4 countries^a^** | | **CAS N=4828, 3 countries^b^** | | **SADC N=19346, 6 countries^c^** | | **ECOWAS N=20789, 5 countries^d^** | |
| --- | --- | --- | --- | --- | --- | --- | --- | --- | --- |
|  |  | OR (95% CI) | p-value | OR (95% CI) | p-value | OR (95% CI) | p-value | OR (95% CI) | p-value |
| **Exposure to risks** | | | | | | | | | |
| Main cooking fuel | Clean fuels | - | - | - | - | - | - | - | - |
|  | Kerosene | 1.85 (0.73, 4.72) | 0.1948 | 1.08 (0.32, 3.58) | 0.9038 | 2.47 (0.58, 10.44) | 0.2187 | 1.37 (0.35, 5.33) | 0.6498 |
|  | Coal, charcoal | 1.53 (0.65, 3.63) | 0.3339 | 1.01 (0.54, 1.88) | 0.9816 | 1.40 (0.87, 2.25) | 0.1707 | 1.19 (0.74, 1.93) | 0.4771 |
|  | Wood | 1.48 (0.62, 3.53) | 0.3767 | 1.11 (0.59, 2.10) | 0.7492 | 1.82 (1.30, 2.55) | <0.0001 | 0.95 (0.61, 1.49) | 0.8387 |
|  | Lower-grade biomass | 1.59 (0.56, 4.48) | 0.3826 | nc. | | 1.31 (0.64, 2.70) | 0.4625 | 0.68 (0.26, 1.80) | 0.4369 |
| Maternal smoking | Yes | 1.53 (1.09, 2.13) | 0.0131 | 1.25 (0.86, 1.82) | 0.2333 | 0.94 (0.74, 1.21) | 0.6439 | 1.34 (1.06, 1.70) | 0.0130 |
|  | No | - | - | - | - | - | - | - | - |
| Time to nearest water source | [coded in 10 min intervals] | 1.01 (1.00, 1.02) | 0.1776 | 1.00 (0.95, 1.04) | 0.9111 | 1.02 (1.01, 1.04) | 0.0042 | 1.01 (1.00, 1.03) | 0.1004 |
| **Non-modifiable risk factors** | | | | | | | | | |
| Child sex | Male | 1.09 (0.97, 1.21) | 0.1439 | 1.04 (0.86, 1.25) | 0.7087 | 1.13 (1.03, 1.24) | 0.0099 | 1.19 (1.08, 1.30) | <0.0001 |
|  | Female | - | - | - | - | - | - | - | - |
| Child age | [years] | 0.95 (0.89,1.01) | 0.1254 | 0.94 (0.85, 1.05) | 0.2548 | 0.92 (0.87, 0.97) | 0.0019 | 0.83 (0.78, 0.88) | <0.0001 |
| Birth order | 1 | - | - | - | - | - | - | - | - |
|  | 2 | 0.96 (0.79, 1.18) | 0.7024 | 0.99 (0.73, 1.35) | 0.9498 | 0.89 (0.77, 1.02) | 0.0996 | 0.83 (0.71, 0.98) | 0.0271 |
|  | ≥3 | 1.00 (0.85, 1.19) | 0.9725 | 0.95 (0.73, 1.23) | 0.6904 | 0.80 (0.71, 0.91) | <0.0001 | 0.92 (0.81, 1.05) | 0.2254 |
| **Household socio-economic status** | | | | | | | | | |
| Shelter index | Low | - | - | - | - | - | - | - | - |
| \|  \| \| --- \| \| | Intermediate | 1.00 (0.87, 1.15) | 0.9564 | 1.06 (0.73, 1.53) | 0.7623 | 1.07 (0.94, 1.22) | 0.2963 | 0.94 (0.79, 1.12) | 0.4781 |
|  | High | 0.84 (0.67, 1.04) | 0.1147 | 0.79 (0.61, 1.03) | 0.0846 | 0.92 (0.78, 1.08) | 0.3052 | 0.87 (0.75, 0.99) | 0.0398 |
| Maternal education | None | 0.84 (0.51, 1.38) | 0.4912 | 0.32 (0.14, 0.71) | 0.0053 | 1.19 (0.71, 1.97) | 0.5091 | 1.06 (0.50, 2.25) | 0.8803 |
|  | Primary | 1.00 (0.62, 1.60) | 0.9850 | 0.54 (0.25, 1.18) | 0.1246 | 1.34 (0.82, 2.19) | 0.2457 | 1.20 (0.56, 2.54) | 0.6403 |
|  | Secondary | 0.85 (0.53, 1.38) | 0.5132 | 0.48 (0.23, 1.02) | 0.0559 | 1.22 (0.75, 1.97) | 0.4221 | 1.32 (0.63, 2.78) | 0.4674 |
|  | Higher | - | - | - | - | - | - | - | - |
| Paternal education | None | 1.87 (1.24, 2.80) | 0.0026 | 0.92 (0.56, 1.52) | 0.7373 | 1.30 (0.89, 1.89) | 0.1752 | 1.19 (0.83, 1.70) | 0.3356 |
|  | Primary | 1.69 (1.15, 2.47) | 0.0072 | 0.96 (0.58, 1.58) | 0.8667 | 1.22 (0.86, 1.72) | 0.2710 | 1.12 (0.78, 1.61) | 0.5287 |
|  | Secondary | 1.68 (1.15, 2.47) | 0.0075 | 0.99 (0.63, 1.54) | 0.9541 | 1.13 (0.80, 1.59) | 0.4827 | 1.16 (0.82, 1.64) | 0.4105 |
|  | Higher | **-** | - | - | - | - | - | - | - |
|  | Never married^e^ | 1.61 (1.02, 2.54) | 0.0424 | 1.19 (0.66, 2.15) | 0.5659 | 1.06 (0.73, 1.54) | 0.7733 | 1.13 (0.67, 1.92) | 0.6479 |
| Stunting | Not stunted | - | - | - | - | - | - | - | - |
|  | Stunted | 0.98 (0.81, 1.19) | 0.8516 | 1.39 (0.98, 1.98) | 0.0682 | 1.19 (1.02, 1.39) | 0.0263 | 1.08 (0.93, 1.27) | 0.3180 |
| Vaccination index | Low | 0.81 (0.65, 1.00) | 0.0472 | 0.85 (0.61, 1.18) | 0.3248 | 0.89 (0.75, 1.05) | 0.1640 | 0.81 (0.70, 0.95) | 0.0079 |
|  | Intermediate | 1.10 (0.89, 1.35) | 0.3878 | 0.89 (0.63, 1.26) | 0.5178 | 0.99 (0.83, 1.19) | 0.9328 | 0.91 (0.78, 1.08) | 0.2805 |
|  | High | - | - | - | - | - | - | - | - |
| **Contextual factors** | | | | | | | | | |
| Rainy season | Yes | 1.94 (0.64, 5.88) | 0.2421 | 0.61 (0.27, 1.38) | 0.2367 | 0.73 (0.49, 1.09) | 0.1205 | 0.68 (0.38, 1.21) | 0.1905 |
|  | No | - | - | - | - | - | - | - | - |
| Geographic location | Countryside | 1.31 (0.83, 2.07) | 0.2428 | 1.08 (0.67, 1.75) | 0.7389 | 0.56 (0.38, 0.85) | 0.0038 | 0.68 (0.42, 1.12) | 0.1331 |
|  | Small city | 0.78 (0.44, 1.40) | 0.4102 | 1.31 (0.78, 2.28) | 0.3101 | 0.57 (0.38, 0.85) | 0.0065 | 0.54 (0.33, 0.90) | 0.0175 |
|  | Town | 1.26 (0.80, 1.97) | 0.3165 | 1.14 (0.71, 1.84) | 0.5807 | 0.61 (0.41, 0.91) | 0.0155 | 0.60 (0.35, 1.02) | 0.0588 |
|  | Capital, large city | - | - | - | - | - | - | - | - |
| **Religion** | Christian | - | - | - | - | - | - | - | - |
|  | Muslim | 0.99 (0.80, 1.22) | 0.9235 | 1.01 (0.75, 1.35) | 0.9717 | 0.97 (0.78, 1.22) | 0.8045 | 0.78 (0.67, 0.91) | 0.0017 |
|  | No or other religion | 1.05 (0.71, 1.57) | 0.7941 | 1.35 (0.98, 1.88) | 0.0702 | 0.92 (0.77, 1.10) | 0.3685 | 0.93 (0.79, 1.09) | 0.3492 |
|  | Unknown | 0.48 (0.22, 1.04) | 0.0623 | nc. | | 0.79 (0.60, 1.04) | 0.0894 | 0.79 (0.57, 1.09) | 0.1447 |
| **Interactions** | | | | | | | | | |
| Main cooking fuel * rainy season | Clean fuels | - | - | - | - | - | - | - | - |
|  | Kerosene | 0.87 (0.23, 3.26) | 0.8317 | 0.81 (0.11, 5.83) | 0.8380 | 0.27 (0.02, 3.38) | 0.3129 | 0.57 (0.09, 3.58) | 0.5488 |
|  | Coal, charcoal | 0.72 (0.23, 2.29) | 0.5814 | 1.25 (0.50, 3.12) | 0.6356 | 1.40 (0.87, 2.25) | 0.1707 | 0.78 (0.42, 1.47) | 0.4457 |
|  | Wood | 0.65 (0.21, 1.99) | 0.4527 | 1.29 (0.55, 3.05) | 0.5616 | 0.86 (0.57, 1.30) | 0.4675 | 0.74 (0.42, 1.33) | 0.3147 |
|  | Lower-grade biomass | 0.62 (0.15, 2.48) | 0.4957 | nc. | | 1.03 (0.31, 3.42) | 0.9586 | 1.45 (0.43, 4.90) | 0.5497 |
| Stunting * child age | Not stunted | - | - | - | - | - | - | - | - |
|  | Stunted | 0.99 (0.89, 1.09) | 0.7931 | 0.82 (0.68, 0.99) | 0.0441 | 0.92 (0.85, 1.00) | 0.0566 | 0.98 (0.90, 1.08) | 0.6957 |
| Vaccination index * child age | Low | 1.02 (0.90, 1.15) | 0.7530 | 1.19 (0.97, 1.45) | 0.0932 | 1.01 (0.91, 1.11) | 0.8882 | 1.11 (1.00, 1.23) | 0.0559 |
|  | Intermediate | 0.95 (0.85, 1.07) | 0.4151 | 1.16 (0.96, 1.40) | 0.1285 | 1.04 (0.94, 1.15) | 0.4751 | 1.09 (0.99, 1.21) | 0.0864 |
|  | High | - | - | - | - | - | - | - | - |
